# Protection against acetaminophen-induced acute liver failure by omentum adipose tissue derived stem cells through the mediation of Nrf2 and cytochrome P450 expression

**DOI:** 10.1186/s12929-016-0231-x

**Published:** 2016-01-19

**Authors:** Yu-Jen Huang, Poda Chen, Chih-Yuan Lee, Sin-Yu Yang, Ming-Tsan Lin, Hsuan-Shu Lee, Yao-Ming Wu

**Affiliations:** Institute of Biotechnology, College of Bioresources and Agriculture, National Taiwan University, Taipei, Taiwan; Department of Surgery, National Taiwan University Hospital, Taipei, Taiwan; Department of Surgery, National Taiwan University Hospital Yun-Lin Branch, Yunlin, Taiwan; Department of Medicine Education & Bioethics Graduate Institute of Medical Education, Bioethics National Taiwan University College of Medicine, Taipei, Taiwan; Department of Internal Medicine, National Taiwan University Hospital and National Taiwan University College of Medicine, Taipei, Taiwan; Department of Surgery, National Taiwan University College of Medicine, Taipei, Taiwan

**Keywords:** Acetaminophen, Acute liver failure, Omentum adipose tissue-derived stem cells, Hepatoprotection

## Abstract

**Background:**

Acetaminophen (APAP) overdose causes acute liver failure (ALF) in animals and humans via the rapid depletion of intracellular glutathione (GSH) and the generation of excess reactive oxygen species (ROS) that damage hepatocytes. Stem cell therapy is a potential treatment strategy for ALF.

**Methods:**

We isolated mesenchymal stem cells (MSCs) from mice omentum adipose tissue-derived stem cells (ASCs) and transplanted them into a mouse model of APAP-induced ALF to explore their therapeutic potential. In addition, we performed in vitro co-culture studies with omentum-derived ASCs and primary isolated hepatocytes to demonstrate the hepatoprotective effect of omentum-derived ASCs on hepatocytes that were subjected to APAP-induced damage.

**Result:**

ASC transplantation significantly improved the survival rate of mice with ALF and attenuated the severity of APAP-induced liver damage by suppressing cytochrome P450 activity to reduce the accumulation of toxic nitrotyrosine and the upregulation of NF-E2-related factor 2 (Nrf2) expression, resulting in an increase in the subsequent antioxidant activity. These effects protected the hepatocytes from APAP-induced damage through the suppression of downstream MAPK signal activation and inflammatory cytokine production.

**Conclusions:**

our results demonstrate that omentum-derived ASCs are an alternative source of ASCs that regulate the antioxidant response and may represent a beneficial therapeutic strategy for ALF.

## Background

Acetaminophen (APAP) is an effective analgesic and antipyretic drug, but it can cause severe liver damage. APAP overdose can result in liver failure, and it is a common cause of acute liver failure (ALF) in Western countries [1]. APAP toxicity is controlled by cytochrome P450, particularly cytochrome P450 subfamily 2E1 (CYP2E1), through the formation of N-acetyl-p-benzoquinoneimine (NAPQI), a highly reactive intermediary and toxic metabolite. This compound subsequently induces oxidative stress and covalently binds to liver proteins, causing cell death and dysfunction. At therapeutic doses, NAPQI conjugates with intracellular glutathione (GSH) and is excreted from the kidney. However, APAP overdose leads to increased NAPQI production, the rapid depletion of GSH and peroxynitrite formation [2]. Excessive NAPQI formation can trigger cell damage via an imbalance in oxidative stress involving high levels of reactive oxygen species (ROS), such as superoxide (O_2_^−^), hydroxyl radicals (OH·), and peroxynitrite, and it leads to low levels of antioxidant enzymes, such as superoxide dismutase (SOD), glutathione peroxidase (GPx), and catalase. Antioxidant defenses can scavenge the excess ROS. For example, SOD can convert O_2_^−^ into H_2_O_2_ and then further convert H_2_O_2_ into H_2_O and O_2_ by GPx and catalase [3]. Consequently, GSH can prevent the covalent binding of toxic metabolites and suppress oxidative stress, which is a potential approach to attenuate APAP toxicity and promote tissue repair/regeneration.

Growing evidence shows that mesenchymal stem cell (MSC) therapy offers advantages for tissue repair and regeneration in animal and clinical studies, based on its immunomodulatory, anti-inflammatory and anti-fibrosis effects and its effects on homing [4]. Adipose tissues have recently been considered as a potential ideal source of MSCs for clinical application because of the minimally invasive procedures needed for collection, the ability to harvest large quantities and their higher potential immunomodulation and anti-inflammatory functions compared with those of other sources [5]. In addition, adipose tissue-derived stem cells (ASCs) exert protective effects and antioxidant properties, enhancing tissue repair and regeneration in animal models of kidney [6] and liver failure [7]. However, the subsequent mechanisms underlying the antioxidant effects and the potential survival benefits are not fully understood. The omentum is the primary and largest intraperitoneal reservoir of adipose tissue. This tissue can be harvested easily during abdominal surgery, and it is even available through a minimally invasive surgery approach. The omentum is therefore a potential source of ASCs; however, the therapeutic potential of omentum-derived ASCs has not yet been evaluated in a disease model. The purpose of this study is to characterize omentum-derived ASCs and to determine whether the transplantation of omentum-derived ASCs exerts therapeutic effects on mice with APAP-induced acute liver failure.

## Methods

### Animals

C57BL/6 mice (male, ~6–8 weeks old) were used as cell donors (hepatocytes and ASCs) and to establish an ALF mouse model for transplantation. All animals were purchased from the National Laboratory Animal Center in Taipei, Taiwan. The mice were housed and handled in accordance with the ethical guidelines for laboratory animal care of the National Taiwan University College of Medicine, and the procedures were approved by the Institutional Animal Care and Use Committee.

### Cells

Mouse hepatocytes were isolated using a 2-step collagenase perfusion method as previously described [8]. ASCs were isolated from mouse omentum adipose tissue, which was cut into small pieces and digested in 0.5 units/ml of collagenase type I (Life Technologies, Paisley, UK) for one hour at 37 °C. After centrifugation at 1500 rpm for 5 min, the pellet was resuspended with PBS (Corning, NY, USA) and seeded into minimum essential medium eagle-alpha modification (α-MEM) supplemented with 1 × antibiotic (Invitrogen, NY, USA) and 10 % fetal bovine serum (FBS, SBI Biological Industries, Belt Haemak, Israel). After 24 h of incubation, the cells were washed with phosphate-buffered saline (PBS), and then the medium was replaced with fresh medium. For all experiments, the cells were used after 3–7 passages and the medium was changed every 3 days. For ASC characterization, the cells were stained with CD31-PE-CY7, CD34-FITC, CD44-PE, CD90-PE (BD Pharmingen, San Diego, CA, USA), CD105-PE (eBioscience, San Diego, CA, USA), and CD29-FITC (BioLegend, San Diego, CA, USA) and then analyzed by flow cytometry (all antibodies were diluted 1:100 with PBS containing 2 % FBS). To evaluate the cell differentiation ability, adipogenic and osteogenic assays were modified according to Sotiropoulon et al. [9]. To evaluate hepatogenic differentiation, the cells were cultured in α-MEM containing 20 ng/ml epidermal growth factor (EGF) and 10 ng/ml fibroblast growth factor (FGF; PeproTech, Rocky Hill, NJ, USA) for 2 days; the medium was then replaced with α-MEM containing 20 ng/ml hepatocyte growth factore (HGF; PeproTech) and 4.9 mM nicotinamine (Sigma-Aldrich, St Louis, MO, USA) for 1 week, followed by treatment with 20 ng/ml oncostatin M (PeproTech), 1 μmol/L dexamethasone (Sigma-Aldrich), and 50 mg/mL insulin-transferrin-selenium (ITS; Gibco, Paisley, UK) that was prepared in α-MEM for 1 week. Glycogen storage was measured by periodic acid-Schiff (PAS) staining (Sigma-Aldrich) [10].

### Acute liver failure model and omentum-derived ASC treatment

Acute liver failure was induced in 8-week-old male C57BL/6 mice. APAP (Sigma-Aldrich) was prepared in saline at 70 °C and maintained in a water bath at 37 °C. Mice were fasted overnight and treated with APAP (600 mg/ kg, intraperitoneally) then randomly divided into two groups: one group was infused with PBS (100 μl/mouse), and the other group was infused with omentum-derived ASCs (10^6^/100 μl/mouse) via tail vein injection at 30 min after APAP treated. For survival, the mice were monitored by 168 h (20 mice/group); At 6 and 24 h after omentum-derived ASCs with/without infusion (10 mice/group) , liver tissue and serum were collected and stored at −80 °C. The liver enzyme (glutamate-oxaloacetate transaminase, GOT; glutamic-pyruvic transaminase, GPT) were evaluated by the Laboratory Animal Center of National Taiwan University Medicine.

### Immunohistology

Liver tissue was fixed in 10 % formaldehyde (Sigma-Aldrich), dehydrated, embedded in paraffin blocks, and sectioned into 5 μm slices. The sections were stained with hematoxylin and eosin (H&E; Sigma-Aldrich) for histology, and the necrosis grade was evaluated in 20 random 100 × images per animal, as described by Liu et al. [11] as follows: “0” indicated normal; “1” indicated necrotic cells in the first cell layer adjacent to the central vein; “2” indicated necrotic cells extending two to three cell layers from the central vein; “3” indicated necrotic cells extending three to six layers from the central vein; “4” indicated necrotic cells extending three to six layers and from one central vein to another; and “5” indicated necrotic cells throughout the section. The sections were deparaffinized and dehydrated for immunohistology, and the endogenous peroxide was inactivated with 3 % hydrogen peroxidase (Sigma-Aldrich). The sections were then blocked with 3 % normal goat serum (DAKO, Glostrup, Denmark) for 1 h, stained with primary antibodies against cytochrome P450 subfamily 2E1 (1:200), 4-hydroxynonenal (1:200, Abcam, Cambridge, MA, USA), or nitrotyrosine (1:50, clone 2A8.2, Millipore, Bedford, MA, USA) for 1 h at 37 °C and then incubated with an horseradish peroxidase (HRP)-detection kit (REAL^TM^ EnVision, DAKO) according to the manufacturer’s protocol.

### Antioxidant enzyme activity assay and GSH content measurement

The activity of the antioxidant enzymes (SOD, GPx, and catalase) and the GSH content were measured according to the manufacturer’s protocol (BioVision, Palo Alto, CA, USA). In brief, the liver tissues from the APAP treatment and the omentum-derived ASC transplantation groups were lysed in PBS by sonication, and the supernatants were collected by centrifugation. For the in vitro experiments, hepatocytes (10^5^) that had been treated with 15 mM APAP were grown in the lower chambers of a six-transwell plate (0.4 μm pore size; BD, Bioscience, San Jose, CA, USA), and omentum-derived ASCs (10^5^) were added to the upper chambers. Twenty-four hours later, the hepatocytes were washed and lysed, and the lysate was collected by centrifugation.

### Western blot

Total proteins were extracted in lysis buffer (300 mM NaCl, 50 mM HEPES pH 7.6, 1.5 mM MgCl_2_, 10 % glycerol, 1 % Triton X-100, 10 mM NaPyrPO_4_, 20 mM NaF, 1 mM EGTA, 0.1 mM EDTA, 1 mM DTT, 1 mM PMSF, and 1 mM Na_4_VO_3_) containing phosphatase inhibitors (all were purchased from Sigma-Aldrich), quantified by protein assay (Bio-Rad, Hercules, CA, USA), separated by 10 % SDS-PAGE (Bio-Rad) and transferred to PVDF membranes (Millipore). After being blocked with 5 % bovine serum albumin (Sigma-Aldrich) in TBS buffer (50 mM Tris–HCl, 150 mM NaCl pH 7.2) with 0.1 % Tween (Sigma-Aldrich) overnight, the membrane was incubated with primary antibody overnight at 4 °C (JNK, phospho-JNK, ERK, phospho-ERK, p38, and phospho-p38 were purchased from Cell Signaling Technology, Danvers, MA, USA 1:2000; cytochrome P450 subfamily 2E1 and 4-hydroxynonenal were purchased from Abcam, 1:1000; and nitrotyrosine and actin were purchased from Millipore at 1:500 and 1:3000, respectively), followed by HRP-conjugated secondary antibody (1:10000, Jackson ImmunoResearch Laboratories, West Grove, PA, USA) for 1 h at room temperature. The protein intensity was detected with electrochemiluminescence (ECL) reagent (Millipore) according to the manufacturer’s protocol. The western blot band intensity was quantified with the ImageJ software according to the manufacturer’s instructions.

### Real-time quantitative PCR

Total RNA was extracted from liver tissue with a Direct-Xol^TM^ RNA MiniPrep kit (Zymo Research, CA, USA). Reverse transcription was performed with an iScript^TM^ cDNA Synthesis kit (Bio-Rad). Q-PCR was performed by using an iCycler real-time detection system and SYBR Green Supermix system (Bio-Rad) according to the manufacturer’s protocol. The primer sequences are provided in Table 1. The relative mRNA levels were determined by Q-PCR and normalized to GAPDH.

**Table 1:** Primer sequences

Primer	Sequence (5‘ → 3’)
HO-1, Forward	CACGCATATACCCGCTACCT
HO-1, Reverse	CCAGAGTGTTCATTCGAGCA
Nrf2, Forward	TTGGAAGGGCTAATGTCCAC
Nrf2, Reverse	CTCCAGCCTCTTGGTTTCTG
NQO1, Forward	TTCTGTGGCTTCCAGGTCTT
NQO1, Reverse	AGGCTGCTTGGAGCAAAATA
IL-1α, Forward	ACATCTTTGACGTTTCAGAGGTT
IL-1α, Reverse	ACGAAGACTACAGTTCTGCCATT
IL-1β, Forward	CCACAGCCACAATGAGTGATACT
IL-1β, Reverse	GAACTCAACTGTGAAATGCCACC
IL-6, Forward	ATTGGAAATTGGGGTAGGAAG
IL-6, Reverse	ACAAGAAAGACAAAGCCAGAGTC
IL-10, Forward	TGGGTTGCCAAGCCTTATCGG
IL-10, Reverse	ACCTGCTCCACTGCCTTGCTC
Cyp2a5, Forward	GGACAAAGAGTTCCTGTCACTGCTTC
Cyp2a5, Reverse	GTGTTCCACTTTCTTGGTTATGAAGTCC
GAPDH, Forward	TGCAGTGGCAAAGTGGAGATT
GAPDH, Reverse	TCGCTCCTGGAAGATGGTGAT

Primers used in quantitative PCR

### ROS, viability and LDH assays

For the ROS assays, the primary hepatocytes were seeded in a 96-well plate (Corning) and treated with various concentrations of APAP (0, 5, 10, 15, 20, and 40 mM) for 24 h. CellROX Deep Red reagent (Life Technologies) was added 30 min before the end point and then washed out with PBS. The ROS intensities were determined by using an ELISA reader (Ex/Em: 644/655 nm, BioTek, Instruments, Inc., Winooski, VT). Cell viability was assessed by the MTT assay (Sigma-Aldrich). Cytotoxicity was determined using the LDH activity assay (BioVision) according to the manufacturer’s instructions.

### Statistical analysis

Values are presented as means ± SEM. Student’s paired t-test or one-way ANOVA followed by Tukey’s test was used for between-group comparisons of the means. The survival analysis was assessed with the SigmaStat software, version 3.5 (Chicago, IL, USA); other analyses were performed with the GraphPad InStat software, version 3 (San Diego, CA, USA). All directional P values were 2-tailed, and a value of .05 or less was considered significant for all tests.

## Result

### Characterization of omentum adipose tissue-derived stem cells (omentum-derived ASCs)

**Fig. 1:**
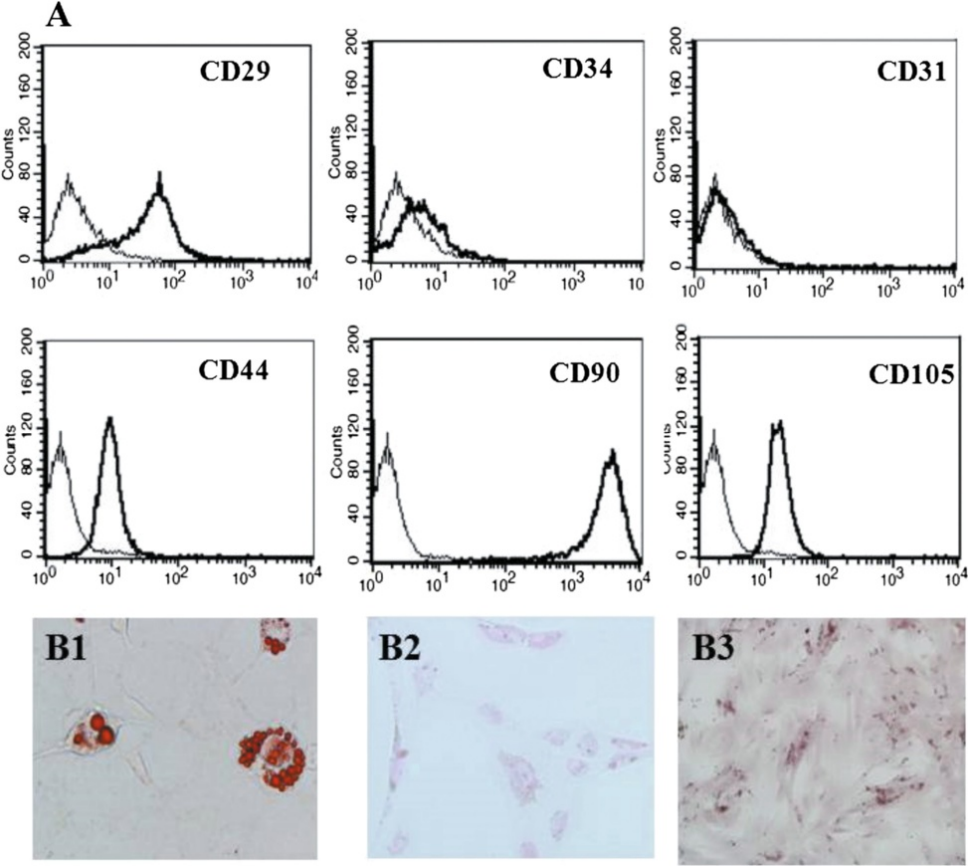
Phenotype and differentiation capacity of omentum-derived ASCs. (**a**) Omentum-derived ASCs were stained with surface markers and analyzed by flow cytometry. (**b**) The cell differentiation ability was assessed in omentum-derived ASCs that were cultured under various differentiation conditions. B1: adipogenic differentiation (Oil Red O staining); B2: hepatogenic differentiation (periodic acid-Schiff staining); and B3: osteogenic differentiation (alizarin staining), 200x

We acquired ASCs from mouse omentum tissue by collagenase digestion. These cells presented fibroblast-like cell morphology and expressed various stem cell markers, including CD29, CD90, CD105, and CD44. The ASCs were negative for CD31 and CD34 (Fig. 1A). We subsequently applied lineage-specific induction factors to assess the differentiation ability of mouse omentum-derived ASCs. The mouse omentum-derived ASCs differentiated into adipocytes, as demonstrated by Oil Red O staining (Fig. 1B1), and osteoblasts, as demonstrated by alizarin staining (Fig. 1B3) after two weeks of induction. Moreover, these cells also differentiated into hepatocytes, as shown by PAS staining, after induction with hepatogenic medium for 16 days (Fig. 1B2). Therefore, the cells that were derived from mouse omentum tissue expressed specific surface markers for MSCs and possessed multi-lineage differentiation ability. These results indicate that omentum is an alternative source to obtain ASCs for subsequent studies or therapy.

### Omentum-derived ASCs protect against APAP-induced liver injury and improve the survival rate of mice with acute liver failure

**Fig. 2:**
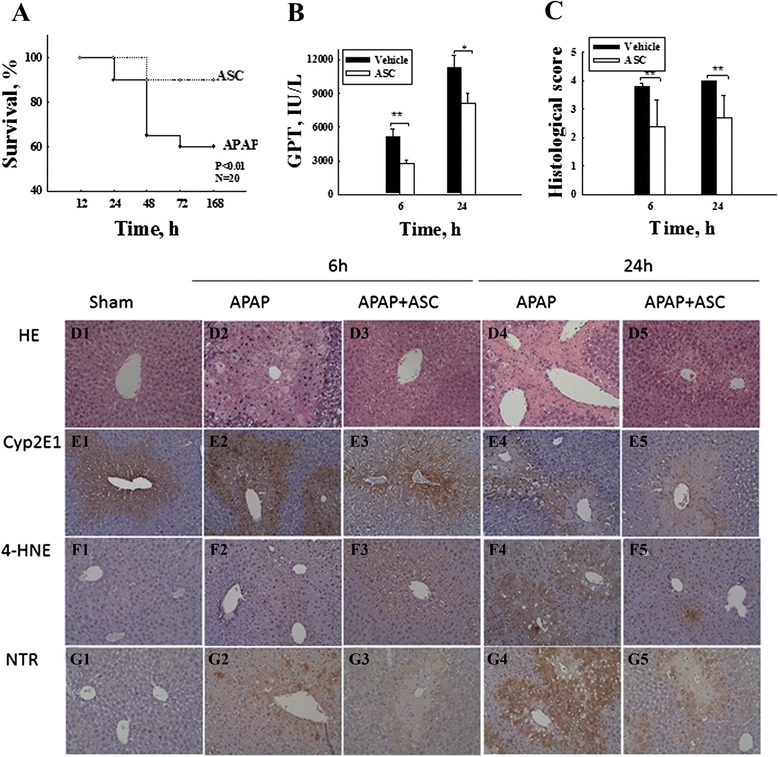
Omentum-derived ASCs prevent APAP-induced hepatotoxicity. Omentum-derived ASCs were transplanted into mice with acute liver failure that had been induced by APAP (600 mg/kg, i.p.). Mouse omentum-derived ASC transplantation significantly improved the survival rate for mice with acute liver failure (*n* = 20 in each group) (**a**) and significantly decreased the liver damage caused by APAP according to both the serum liver enzyme (GPT) levels (**b**) and the tissue histological grade (**c**). The data are expressed as the means ± SEM, n ≥ 5, *P < 0.05, **P < 0.01. (D) H&E staining shows that APAP caused severe centrilobular necrosis (D2: 6 h after APAP; D4: 24 h after APAP). Omentum-derived ASC transplantation decreased the severity of liver damage caused by APAP (D3: 6 h after APAP + omentum-derived ASC treatment; D5: 24 h after APAP + omentum-derived ASC treatment). Immunohistological staining shows that APAP treatment induced the expression of CYP2E1 (E2-E5), 4-HNE (F2-F5), and NTR (G2-G5), which were suppressed after omentum-derived ASC transplantation. CYP2E1: cytochrome P450 2E1; 4-HNE: 4-hydroxynonenal; and NTR: nitrotyrosine. Magnification, 200x

To explore the therapeutic effects of omentum-derived ASCs on ALF, we used the ALF mouse model induced by APAP. Eight of 20 mice were died within 24–72 h after 600 mg/kg of APAP injection (60 % of survival rate), only 10 % of mice were died after omentum-derived ASC treatment (90 % of survival rate vs. control group). The overall differenece in survival rate between groups with and without omentum-derived ASC was significant (*P* ≤ 0.01, Fig. 2A). The severity of APAP-induced liver damage was evaluated by measuring the plasma liver enzyme level and histological necrosis score at 6 and 24 h after APAP injection. The plasma GPT level decreased significantly at both time points after treatment with omentum-derived ASCs (5097 ± 703 IU/L compared with 2787 ± 260 IU/L at 6 h, *P* < 0.01, and 11259 ± 1159 IU/L compared with 8141 ± 910 IU/L at 24 h, *P* < 0.05) (Fig. 2B). In addition, liver histological staining showed extensive necrosis with inflammation and ballooning in the centrolobular region at 6 h after APAP exposure (Fig. 2D2) (histological necrosis score: 3.8) and extremely severe necrosis (score: 4) at 24 h after APAP administration (Fig. 2D4). The transplantation of omentum-derived ASCs decreased the area of cell necrosis (Fig. 2D3, D5) and significantly attenuated the histological necrosis score severity (2.5 at both 6 and 24 h, *P* < 0.01, Fig. 2C). These results showed that omentum-derived ASCs have therapeutic effects on APAP-induced liver toxicity.

### Omentum ASC transplantation prevents GSH depletion and enhances antioxidant enzyme activity

**Fig. 3:**
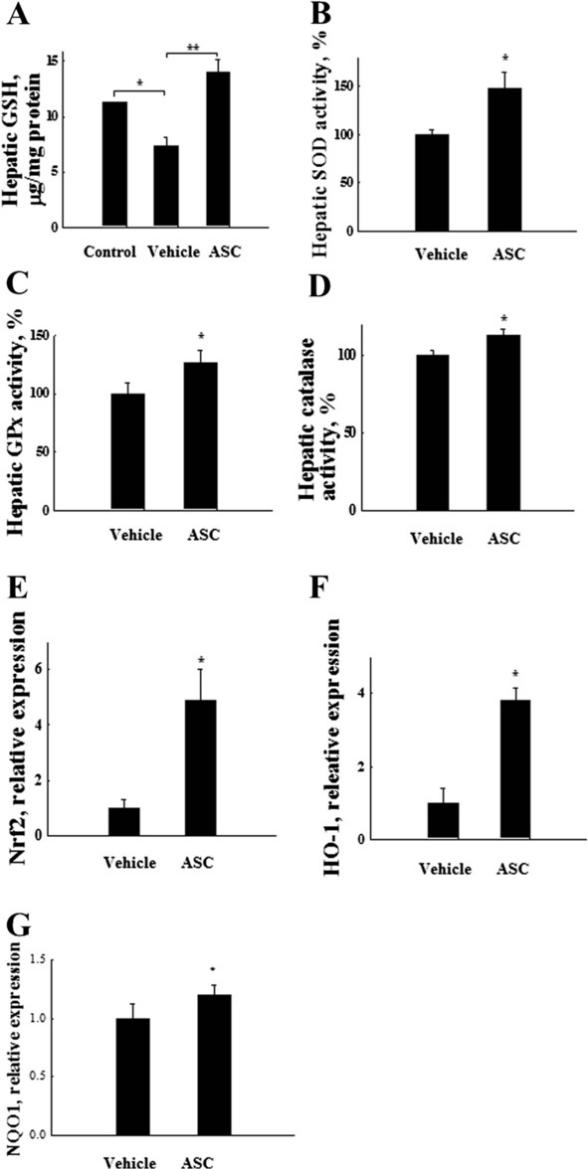
Omentum-derived ASCs protect the liver against APAP-induced injury via the increased activity of antioxidant enzymes. Liver tissue samples were collected 6 h after omentum-derived ASC transplantation. The lysate was assessed for GSH content and antioxidant enzyme activity. The (**a**) GSH content and antioxidant enzyme activities of (**b**) SOD, (**c**) GPx, and (**d**) catalase increased significantly after omentum-derived ASC transplantation. The expression of (**e**) HO-1, (**f**) Nrf2 and (**g**) NQO1 are increased significantly 24 h after omentum-derived ASC transplantation. The data are expressed as the means ± SEM, n ≥ 5, *P < 0.05, **P < 0.01

Next, we studied whether the hepatoprotective effect of omentum-derived ASCs was associated with antioxidant activity in APAP-induced liver injury. The GSH levels dramatically declined after 6 h of APAP exposure, indicating that the high oxidative stress was induced after APAP injection. However, the depletion of the liver GSH content significantly improved 6 h after omentum-derived ASC transplantation, with higher hepatic GSH content than the content after APAP administration (*P* < 0.01) (Fig. 3a). Moreover, APAP overdose caused oxidative stress that resulted from an imbalance between oxidant generation and antioxidant defense. Transplantation with omentum-derived ASCs significantly increased hepatic antioxidant enzyme activity. SOD activity was increased by 47 %, GPx activity was enhanced by 28 %, and catalase activity increased by as much as 12 % compared with the activity levels in the APAP administered group (Fig. 3b–d). Furthermore, Nrf2 is a master regulator of the transcriptional activation of genes related to the antioxidant defense system, and it controls downstream heme oxygenase-1 (HO-1) expression. We examined Nrf2, HO-1 and NADPH quinone oxidoreductase 1 (NQO1) gene expression by Q-PCR and found that these gene expression significantly increased in the omentum-derived ASC group compared with the APAP-treated group (Fig. 3e–g). These results suggested that omentum-derived ASCs protect against APAP toxicity by enhancing antioxidant defense and reducing GSH depletion.

### Omentum-derived ASCs affect the metabolism of APAP

**Fig. 4:**
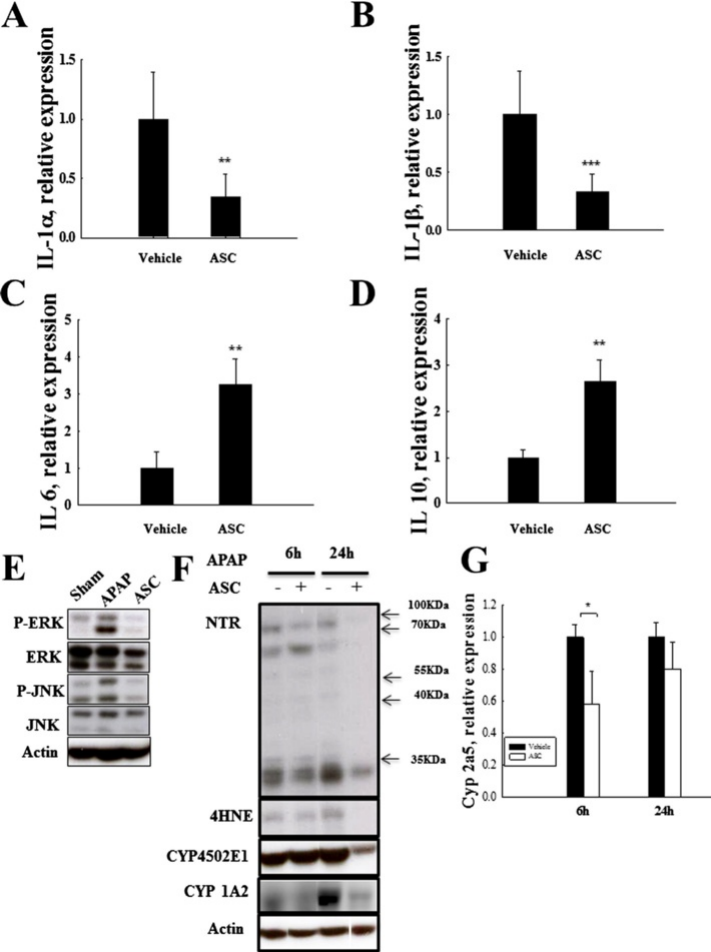
Omentum-derived ASC transplantation attenuated APAP-induced inflammation, and MAPK signaling activation. Omentum-derived ASC transplantation decreased the expression of the pro-inflammatory cytokines (**a**) IL-1α and (**b**) IL-1β and increased the expression of anti-inflammatory cytokines (**c**) IL-6 and (**d**) IL-10 to a significant degree. Omentum-derived ASC transplantation suppressed the MAPK signaling pathway activation induced by APAP treatment. **e** Liver tissue was collected 6 h after omentum-derived ASC transplantation into APAP-treated mice and used for RT-PCR (**a**, **b**, **c** and **d**) and western blot (**e**) analysis. (**f**) CYP 450 2E1, CYP 450 1A2, NTR, and 4-HNE protein expression by western blot analysis. (**g**) CYP2A5 gene expression by RT-PCR. The data are expressed as the means ± SEM, n ≥ 5, **P < 0.01, ***P < 0.001

We further studied whether the transplantation of omentum-derived ASCs affected APAP metabolism. CYP2E1 is an important cytochrome enzyme that is responsible for the toxic metabolism of APAP to NAPQI, which depletes GSH. Additionally, CYP2E1 binds to vital proteins and causes cell death. Immunohistology revealed that CYP2E1 was strongly expressed 6 h after APAP treatment but only weakly expressed in the omentum-derived-ASC-treated group (Fig. 2E). The transplantation of omentum-derived ASCs markedly reduced the expression of CYP2E1 protein 24 h after APAP treatment (Fig. 4f). Beside, other forms of cytochrome P450 expression were revealed that CYP1A2 protein was decreased significantly at 24 h (Fig. 4f) and Cyp2a5 gene level was reduced significantly at 6 h after omentum-derived ASCs transplantation (Fig. 4g). Oxidative stress induces lipid peroxidation to produce 4-HNE, and 4-HNE is considered a biomarker of lipid peroxidation. After 24 h of APAP exposure, the liver sections showed increased 4-HNE-positive cells that were localized in the centrilobular area (Fig. 2F4). Immunohistology and western blots showed that the 4-HNE expression in the omentum-derived ASC transplantation group was lower than that of the APAP group (Figs. 2F4 and 4f). Moreover, APAP overdose caused nitrotyrosine protein formation in the centrilobular region. Protein nitration, which is a marker of oxidative stress that is caused by peroxynitrite, occurs through the rapid reaction of superoxide with nitric oxide. Nitrotyrosine protein increased strongly in the centrilobular regions 24 h after APAP injection but was only weakly expressed in the omentum-derived ASC treatment group (Figs. 2G and 4f). Therefore, the transplantation of omentum-derived ASCs suppressed cytochrome P450 activity by decreasing the production of the toxic APAP metabolite NAPQI. This response led to the decreased consumption of GSH and decreased oxidative stress, protecting hepatocytes from APAP-induced cell damage.

### Omentum ASCs attenuate MAPK signal activation and the inflammatory response in vivo

Accumulated toxic APAP metabolites can generate oxidative stress and subsequently activate mitogen-activated protein kinases (MAPK) signaling and inflammatory cytokine production to induce further cell damage. The gene expression of the pro-inflammatory cytokines IL-1α (Fig. 4a) and IL-1β (Fig. 4b) decreased significantly in the omentum-derived ASC group 6 h after APAP injection. The gene expression of the anti-inflammatory cytokines IL-6 (Fig. 4c) and IL-10 (Fig. 4d) increased significantly in the omentum-derived ASC group 6 h after APAP injection. MAPK signaling pathways play critical roles in mediating APAP hepatotoxicity. The phosphorylation of the ERK and JNK proteins (pERK and pJNK) increased 6 h after APAP injection, but this response was suppressed in the omentum-derived ASC group (Fig. 4e). These results showed that the protective effect of omentum-derived ASCs against APAP toxicity also suppressed MAPK activation and attenuated the inflammatory response.

### Hepatoprotective effect of omentum ASCs on APAP-induced damage in isolated hepatocytes

**Fig. 5:**
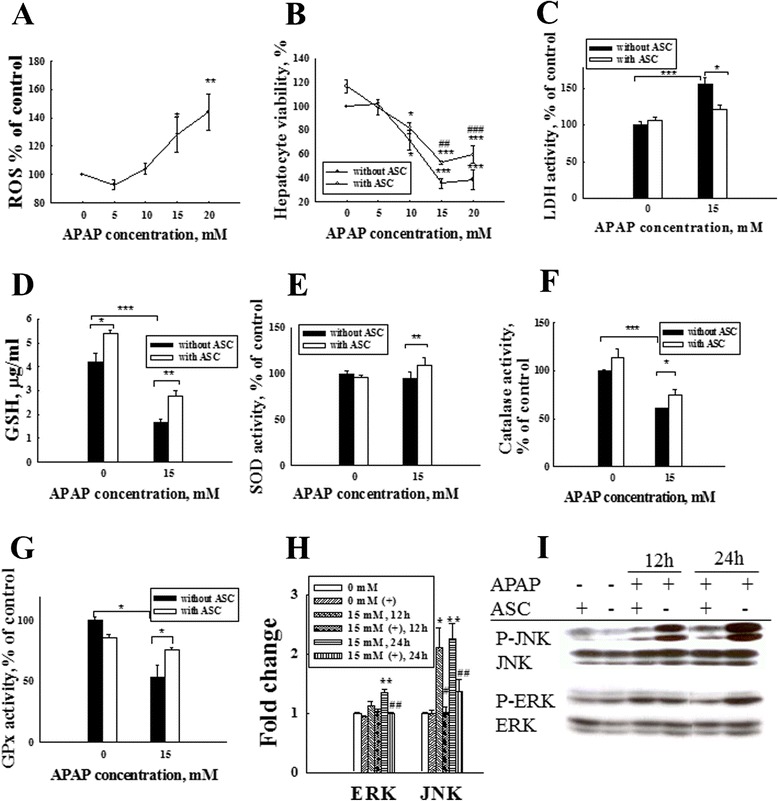
Omentum-derived ASCs protect primary isolated hepatocytes from APAP-induced toxicity. **a** Primary isolated hepatocytes were exposed to various concentrations of APAP for 24 h, and the intracellular ROS levels were measured by Cell ROX assay. The intracellular ROS levels increased significantly after APAP treatments at concentrations that exceeded 15 mM (**a**). Hepatocyte viability decreased significantly after treatment with the same APAP concentration (**b**). We chose 15 mM APAP for the subsequent in vitro studies. Hepatocytes were treated with APAP and co-cultured with/without omentum-derived ASCs in a transwell co-culture system. The viability of the APAP-treated hepatocytes improved significantly after 24 h of co-culture with omentum-derived ASCs, as shown by the MTT assay (**b**). LDH that was released into the culture medium decreased significantly after co-culture with omentum-derived ASCs (**c**). The GSH content of APAP-treated hepatocytes increased significantly after 24 h of co-culture with omentum-derived ASCs (**d**). The antioxidant enzyme (SOD, catalase, and GPx) activity also increased significantly after co-culture with omentum-derived ASCs (**e**, **f**, **g**). The expression of phosphorylated MAPK signaling pathway proteins (ERK/JNK) in APAP-treated hepatocytes decreased significantly after 24 h of co-culture with omentum-derived ASCs (**h**, **i**). The quantification of the phosphorylated proteins in each lane was normalized to the total protein levels. The data are expressed as the means ± SEM, n ≥ 5, *P < 0.05, **P < 0.01, and ***P < 0.001

To clarify whether the omentum-derived ASCs directly protect against APAP toxicity in primary mouse hepatocytes, we isolated primary hepatocytes and exposed them to various APAP concentrations to measure their ROS production by CellROX assay. The ROS generation was dose dependent and increased significantly in response to 15 and 20 mM of APAP exposure (Fig. 5a). The viability of the hepatocytes decreased significantly to 70 % of the pretreatment level after treating with 10 mM APAP, and it decreased dramatically to 35 % after treating with 15 mM APAP (Fig. 5b). We chose to administer 15 mM APAP during the subsequent in vitro co-culture mechanistic studies because no differences in viability were observed in omentum-derived ASCs at this APAP concentration (data not shown). The viability of APAP-treated hepatocytes increased significantly after co-culture with omentum-derived ASCs compared with APAP exposure alone at 24 h (52 % compared with 35 % at 15 mM APAP, *P* < 0.001). The LDH release after APAP treatment was higher than that of the control hepatocytes (*P* < 0.001), but it was reduced in the omentum-derived ASC co-culture group (*P* < 0.05) (Fig. 5c). These results show that omentum-derived ASCs significantly increased the viability and markedly reduced the LDH release in hepatocytes that were treated with APAP. NAPQI is a toxic metabolite of APAP that is able to cause GSH depletion by covalently binding to hepatic GSH. The GSH content of omentum-derived ASCs co-cultured hepatocytes increased to 5.4 μg/ml (compared with 4.7 μg/ml in normal hepatocytes, *P* < 0.05). The GSH content in cultured hepatocytes decreased to 1.67 μg/ml after APAP exposure but then increased to 2.78 μg/ml in APAP-treated hepatocytes that were co-cultured with omentum-derived ASCs (*P* < 0.01) (Fig. 5d). These results show that omentum-derived ASCs increased the hepatic GSH content, which could attenuate toxic NAPQI formation. Furthermore, the activity of antioxidant enzymes in hepatocytes significantly decreased after APAP exposure for 24 h (catalase activity: 64.9 %, Fig. 5f and GPx activity: 53.9 %, Fig. 5g). The activity of antioxidant enzymes in APAP-treated hepatocytes increased significantly after co-culture with omentum-derived ASCs (SOD and catalase activity: 10 %, *P* < 0.05 and GPx activity: 20 %, *P* < 0.05). Finally, we studied whether MAPK pathways were involved in the protection of omentum-derived ASCs against APAP hepatotoxicity. The levels of JNK and ERK phosphoproteins were significantly increased in hepatocytes at 12 h and 24 h after APAP exposure but were suppressed after co-culture with omentum-derived ASCs (Fig. 5h, i). Therefore, the activation of the JNK/ERK pathway by APAP metabolites was diminished by omentum-derived ASC treatment.

## Discussion

**Fig. 6:**
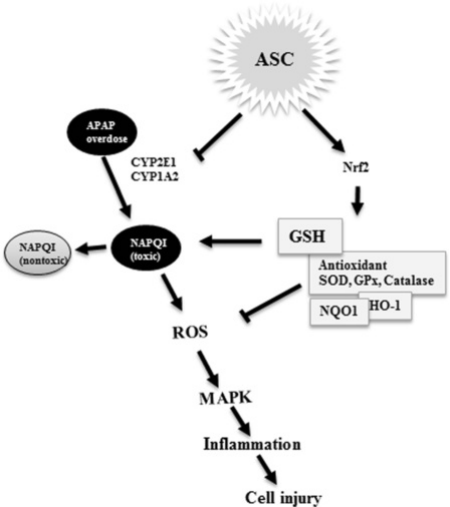
Schematic of potential targets for omentum-derived ASC protection against APAP-induced hepatotoxicity. APAP overdose is metabolized by CYP2E1 and CYP1A2 to form the toxic metabolite NAPQI, which rapidly consumes intracellular GSH and causes ROS generation followed by the activation of the MAPK pathway, leading to inflammation and cell death. However, omentum-derived ASCs suppress CYP2E1 and CYP1A2 activity and activate Nrf2 expression to reduce the formation of the toxic metabolite NAPQI and subsequent ROS generation, resulting in attenuated APAP-induced toxicity. APAP: acetaminophen; ASC: adipose tissue-derived stem cells; CYP2E1: cytochrome P450 subfamily 2E1; CYP1A2: cytochrome P450 subfamily 1A2; NAPQI: N-acetyl-p-benzoquinoneimine; GSH: glutathione; ROS: reactive oxygen species; MAPK: mitogen-activated protein kinases; Nrf2: NF-E2-related factor 2; SOD: superoxide dismutase; GPx: glutathione peroxidase; NQO1: NADPH quinone oxidoreductase 1; HO-1: heme oxygenase-1

In this study, we investigated whether omentum-derived ASC therapy could rescue APAP-induced acute liver failure. We found that the transplantation of omentum-derived ASCs significantly improved the survival of mice with APAP-induced acute liver failure. Additionally, we showed that omentum-derived ASCs scavenged ROS through the upregulation of Nrf2 activation, and they decreased toxic peroxynitrite formation by suppressing cytochrome P450 expression. Finally, omentum-derived ASCs attenuated the subsequent inflammatory response and the activation of MAPK signaling to protect against APAP-induced hepatocyte death (Fig. 6). We deduce that mechanism that mediate the protectives effect of omentum-derived ASCs are immunosuppressing, which downregulation of proinflammatory cytokines (IL-1α, IL-1β) and upregulation of anti-inflammatory (IL-6, IL-10). And, paracrine properties of MSC to improve liver function and guide MSCs homes to injury site mediated by their expression of growth factors and cytokines. It is worth nothing that protective effects of MSCs are antioxidant. Based on our observation, omentum-derived ASCs are highly resistant to APAP-induced death and scavenge ROS, increase intracellular GSH levels and antioxidant enzyme activity mediated by Nrf2 expression.

APAP toxicity is mediated by cytochrome P450 metabolism to NAPQI, which causes hepatic GSH depletion, lipid peroxidation, and nitrotyrosine protein accumulation leading to cell death. It has been suggested that an effective strategy for preventing APAP hepatotoxicity is to inhibit CYP2E1 activity [12, 13]. Lee also showed that CYP2E1-knockout mice were less susceptible to APAP toxicity [14, 15]. Our immunohistology results showed that CYP2E1 expression decreased significantly in the omentum-derived-ASC-treated group. In addition, CYP1A2 and CYP2A5 have metabolic activity toward APAP. We also found both CYP1A2 and CYP2A5 were inhibited after omentum-derived ASCs transplantation. Therefore, suppression of cytochrome P450 activity contributed to the hepatoprotective effect of omentum-derived ASCs. APAP-induced hepatotoxicity consists of a metabolic phase and an oxidative phase. During the metabolic phase, the metabolite NAPQI causes GSH depletion and covalent binding to liver proteins, which triggers cell dysfunction and generates ROS that induce lipid peroxidation and interfere with antioxidant defense mechanisms during the oxidative phase [16]. Nrf2 is a transcription factor that regulates the expression of phase II enzymes and transports protein metabolites to protect cells against oxidative stress [17]. Chan et al. [18] demonstrated that *Nrf2*^*−/−*^ mice are more sensitive to APAP toxicity and have lower levels of liver GSH. MSCs can also act as an antioxidant to regulate the oxidative microenvironment [19–21]. In a recent study [7, 22] on MSC antioxidant ability, MSC transplantation reportedly reduced oxidative stress by supplying GSH in the liver of animals with APAP overdose. Thus, our results showing protective effects were consistent with their finding. Furthermore, key findings in the current study include that omentum-derived ASCs were essential to upregulating Nrf2 and that they inhibited cytochrome P450 expression to protect cells against APAP toxicity. It is possible that MSCs express CD44 markers, which reportedly activate the Nrf2 pathway to protect against APAP toxicity [16, 23, 24]. We successfully isolated ASCs from the omentum and demonstrated their MSC properties, and the results revealed that the omentum-derived ASCs significantly increased Nrf2 expression to activate antioxidant enzyme activity (SOD, GPx, and catalase) and cellular GSH synthesis. Omentum-derived ASCs were able to scavenge excess ROS by activating the Nrf2 pathway, leading to increased GSH synthesis and enhanced antioxidant defense. N-acetylcysteine (NAC) protects against APAP hepatotoxicity by increasing the intracellular GSH content that is available to conjugate to NAPQI in animal experiments [25], indicating a role for these cells as a potential therapy for APAP-induced acute liver failure in clinical practice [26]. However, the limitations of NAC therapy include a short therapeutic time window, adverse gastrointestinal effects and an anaphylactoid reaction [1]. Consequently, the antioxidative effect of omentum-derived ASCs offers another therapeutic approach to protect against APAP hepatotoxicity in clinical practice.

The toxic metabolites of APAP damage hepatocytes and cause the release of inflammatory mediators, particularly IL-1α and IL-1β [27], which induce further cell damage. MSCs also exhibit immunomodulatory properties [28, 29]. Our results show that omentum-derived ASCs significantly suppressed the release of pro-inflammatory cytokines (IL-1α and IL-1β) and increased the release of anti-inflammatory cytokines (IL-6 and IL-10). The immunomodulation effect of omentum-derived ASCs also contributed to the efficiency of protection against APAP-induced hepatotoxicity. These inflammatory mediators are regulated by MAPK signal transduction, which plays a central role in cell survival, proliferation, apoptosis, and inflammation [30]. One potential anti-inflammatory therapeutic strategy is to suppress the activation of the MAPK to reduce pro-inflammatory cytokine release and promote anti-inflammatory cytokine production [7, 31]. Our results showed that omentum-derived ASCs also have an immunomodulatory effect by regulating the MAPK pathway.

## Conclusions

In conclusion, our results show that ASCs can be obtained from omentum adipose tissue, and they possess antioxidant and anti-inflammatory properties that provide protection against APAP-induced hepatotoxicity. Thus, omentum-derived ASCs have potential as an alternative source for cell therapy, and may be is an effective therapeutic strategy for APAP-induced liver failure in clinical practice.

## Abbreviations

APAP: acetaminophen
GSH: glutathione
ROS: reactive oxygen species
ASC: adipose tissue-derived stem cell
ALF: acute liver failure
SOD: superoxide dismutase
GPx: glutathione peroxidase
CYP2E1: cytochrome P450 subfamily 2E1
CYP1A2: cytochrome P450 subfamily 1A2
CYP2A5: cytochrome P450 subfamly 2A5
Nrf2: NF-E2 related factor 2
NQO1: NADPH quinone oxidoreductase
HO-1: heme oxygenase-1
MAPK: mitogen-activated protein kinase
NAPQI: N-acetyl-p-benzoquinoneimine
O_2_^−^: superoxide
OH^.^: hydroxyl radicals
MSC: mesenchymal stem cell
NAC: N-acetyl cysteine
